# 525. Atovaquone for Treatment of COVID-19 (Ataq COVID-19) Trial

**DOI:** 10.1093/ofid/ofab466.724

**Published:** 2021-12-04

**Authors:** Mamta K Jain, Mamta K Jain, Hesham Sadek, James de Lemos, Darren mcGuire, Colby Ayers, Jennifer L Eiston, Claudia Lucas, Dena Kamel, Xilong Li, Barbine Agbor Agbor, Noelle Williams, John Schoggins

**Affiliations:** UT Southwestern Medical Center, Dallas, TX

## Abstract

**Background:**

Our group performed an in-silico screen to identify FDA approved drugs that inhibit SARS-C0V-2 main protease (Mpro), followed by in vitro viral replication assays, and in vivo pharmacokinetic studies in mice. These studies identified atovaquone as a promising candidate for inhibiting viral replication.

**Methods:**

Enrolled patients were randomized in a 2:1 fashion to atovaquone 1500 mg twice daily versus matched placebo. Patients received standard of care treatment including remdesivir, dexamethasone, or convalescent plasma as deemed necessary by the treating team. Patients agreed to allow collection of saliva at baseline and twice a day while hospitalized or up to 10 days. Saliva was collected and RNA extracted for viral load (VL) measurement by Real-time PCR. Our primary outcome was to examine the between group differences in log transformed VL(copies/mL) using generalized linear mixed-effect models of repeated measures from all samples. Additional analysis of Atovquone plasma concentrations were examined and correlated with viral load and body mass index (BMI).

**Results:**

Of the 61 patients enrolled; 41 were received atovaquone and 19 placebo. Overall the population was predominately male Hispanic with a mean age of 51 years. The two groups were balanced (Table 1) with regard to age, gender, race, co-morbidities, days from onset of symptoms, baseline oxygen requirements, and receipt of COVID-19 specific standard of care treatment. A higher proportion with diabetes was noted in the Atovaquone arm. The log_10_ VL was 5.25 copies/mL vs. 4.79 copies/mL at baseline in the atovaquone vs. placebo group. Although there was a decrease in VL over time, there was no differences between the atovaquone plus standard of care arm versus the standard of care arm (Figure 1). Additional analysis of atovaquone plasma concentration demonstrated a wide variation in atovaquone levels, inverse association between atovaquone levels and BMI (rho -0.44, p=0.03), and Day 5 concentrations and VL (rho -0.54, p=0.005).

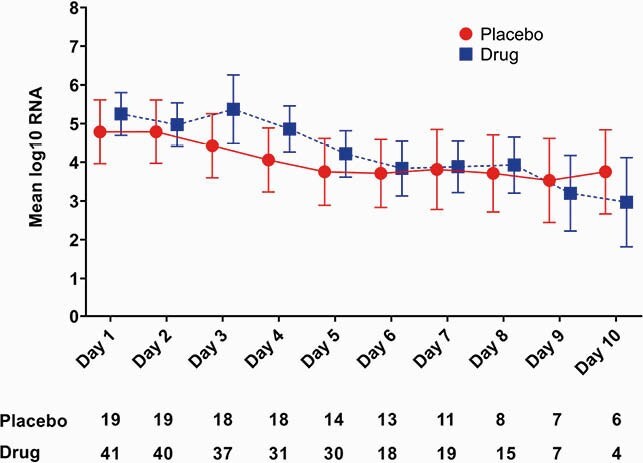

Figure 1. Mean viral load of COVID-19 over time of atovaquone (blue) vs. placebo (red).

Table 1. Baseline characteristics

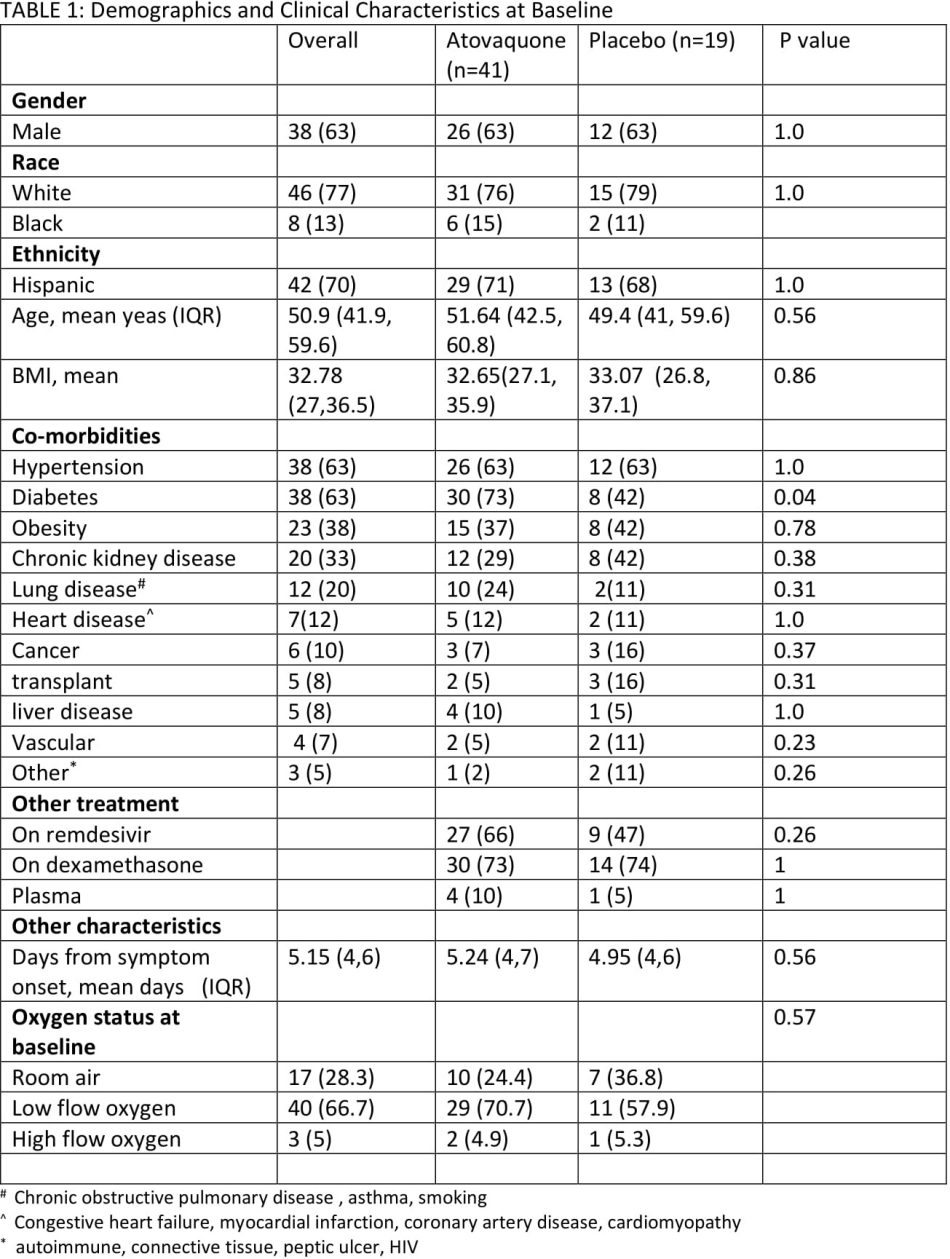

**Conclusion:**

Although atovaquone showed promising in vitro antiviral properties for COVID-19, in this pilot study we did not detect a change in VL in patients who received atovaquone compared to placebo, possibly due to failure of patients achieve adequate drug levels.

**Disclosures:**

**Mamta K. Jain, MD, MPH**, Gilead Sciences Inc. (Individual(s) Involved: Self): Research Grant or Support, Scientific Research Study Investigator; GlaxoSmithKline (Individual(s) Involved: Self): Scientific Research Study Investigator; Merck (Individual(s) Involved: Self): Scientific Research Study Investigator; Vasgene (Individual(s) Involved: Self): Scientific Research Study Investigator

